# Comparative assessment of phenotypic markers in patients with chronic inflammation: Differences on *Bifidobacterium* concerning liver status

**DOI:** 10.1111/eci.14339

**Published:** 2024-10-28

**Authors:** Lourdes Chero‐Sandoval, Andrea Higuera‐Gómez, María Martínez‐Urbistondo, Raquel Castejón, Susana Mellor‐Pita, Víctor Moreno‐Torres, Daniel de Luis, Amanda Cuevas‐Sierra, J. Alfredo Martínez

**Affiliations:** ^1^ Precision Nutrition and Cardiometabolic Health, IMDEA‐Food Institute (Madrid Institute for Advanced Studies) Campus of International Excellence (CEI) UAM+CSIC Madrid Spain; ^2^ Department of Endocrinology and Nutrition, University Clinical Hospital University of Valladolid Valladolid Spain; ^3^ Internal Medicine Service Puerta de Hierro Majadahonda University Hospital Madrid Spain; ^4^ Health Sciences School and Medical Centre International University of the Rioja (UNIR) Madrid Spain; ^5^ Centre of Endocrinology and Nutrition University of Valladolid Valladolid Spain; ^6^ CIBERobn Physiopathology of Obesity and Nutrition Institute of Health Carlos III (ISCIII) Madrid Spain

**Keywords:** *Bifidobacterium*, Fatty Liver Index, gut microbiota, hepatic status, systemic lupus erythematosus

## Abstract

**Background:**

The relationship between systemic lupus erythematosus (SLE) and low‐grade metabolic inflammation (MI) with the microbiota is crucial for understanding the pathogenesis of these diseases and developing effective therapeutic interventions. In this context, it has been observed that the gut microbiota plays a key role in the immune regulation and inflammation contributing to the exacerbation through inflammatory mediators. This research aimed to describe similarities/differences in anthropometric, biochemical, inflammatory, and hepatic markers as well as to examine the putative role of gut microbiota concerning two inflammatory conditions: SLE and MI.

**Methods:**

Data were obtained from a cohort comprising adults with SLE and MI. Faecal samples were determined by 16S technique. Statistical analyses compared anthropometric and clinical variables, and LEfSe and MetagenomeSeq were used for metagenomic data. An interaction analysis was fitted to investigate associations of microbiota with fatty liver index (FLI) depending on the inflammatory condition.

**Results:**

Participants with low‐grade MI showed worse values in anthropometry and biochemicals compared with patients with SLE. The liver profile of patients with MI was unhealthier, while no relevant differences were found in most of the inflammatory markers between groups. LEfSe analysis revealed an overrepresentation of Bifidobacteriaceae family in SLE group. An interactive association between gut *Bifidobacterium* abundance and type of disease was identified for FLI values, suggesting an effect modification of the gut microbiota concerning liver markers depending on the inflammatory condition.

**Conclusion:**

This study found phenotypical and microbial similarities and disparities between these two inflammatory conditions, evidenced in clinical and hepatic markers, and showed the interactive interplay between gut *Bifidobacterium* and liver health (measured by FLI) that occur in a different manner depending on the type of inflammatory disease. These results underscore the importance of personalized approaches and individual microbiota in the screening of different inflammatory situations, considering unique hepatic and microbiota profiles.

## Graphical abstract

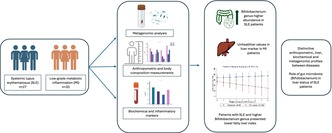

**Key Points**

- Participants with low‐grade metabolic inflammation (MI) presented worse values in anthropometrics, body composition, biochemical and hepatic variables comparing with participants with an autoimmune disease such as systemic lupus erythematosus (SLE), but no relevant differences for inflammatory markers were found.
- Gut microbiota presented some differences between SLE and low‐grade MI. Patients with SLE overrepresented bacteria from Actinobacteria class, Bifidobacteriales order, Bifidobacteriaceae family, *Bifidobacterium* genus and *Bifidobacterium adolescentis* specie, in contrast with low‐grade MI. *Bifidobacterium* genus was confirmed to be higher abundant in SLE using MetagenomeSeq analysis.
- The abundance of *Bifidobacterium* was significantly related with lower Fatty Liver Index (FLI) values in SLE participants, but not in low‐grade MI, suggesting that the relationship between gut microbiota composition and FLI varies depending on the type of inflammatory condition.

## 1 INTRODUCTION

Autoimmune diseases and some metabolic disorders are medical conditions, involving shared and distinctive inflammatory features, whose interactions and associations have increased interest for precision medicine purposes.^1^ Autoimmune diseases are characterized by impaired immune responses directed against self‐antigens, resulting in tissue damage and cellular dysfunctions with associated inflammatory processes.^2^ These conditions affect multiple organ systems, including diseases such as systemic lupus erythematosus (SLE), rheumatoid arthritis, multiple sclerosis, type 1 diabetes mellitus, and autoimmune thyroid diseases.^3^ On the other hand, metabolic disorders involve dysfunctions in biochemical and physiopathological markers gathered as a cluster of conditions and disease manifestations including obesity, insulin resistance, dyslipidemia, hypertension, low‐ grade inflammation and metabolic syndrome (MetS).^4^, ^5^ The pathophysiological features of SLE and metabolic inflammation include immune dysfunction, chronic inflammation, and insulin resistance. In SLE, immune dysfunction is manifested by abnormal activation of B and T lymphocytes, resulting in the production of tissue‐damaging autoantibodies.^6^ This phenomenon is also observed in metabolic inflammation, where activation of the innate immune system contributes to aggravating dysfunction.^7^, ^8^ Chronic inflammation, characterized by elevated levels of proinflammatory cytokines such as interleukin‐6 (IL‐6) and tumour necrosis factor‐ α (TNF‐α), is common in both conditions and aggravates disease progression.^9^ In addition, insulin resistance, which increases cardiovascular risk, is associated with systemic inflammation and metabolic disturbances in SLE patients,^10^ which gives support to delve deeper into the pathophysiology features and including microbiota participation as a putative bidirectional factor associated to inflammation.^11^ Emerging evidence suggests a significant interconnection between these two groups of disorders, where immunological and metabolic factors converge to influence pathogenesis and clinical course.^12^, ^13^

In addition, consistent evidence has shown complex relationships between inflammatory diseases and the intestinal microbiota, which emerge as a crucial determinant of health outcomes.^14^ In this sense, dysbiosis, defined as an imbalance in gut microbial communities, plays an important role in the initiation and perpetuation of chronic inflammatory responses, linking to various immune‐mediated inflammatory diseases, such as SLE and low‐grade metabolic inflammation (MI). This microbial imbalance reduces bacterial diversity and alters the production of short‐chain fatty acids (SCFAs), such as butyrate, affecting immune regulation and promoting inflammation.^15^, ^16^, ^17^

In this sense, the genus *Bifidobacterium* is considered essential in the gastrointestinal tract,^18^, ^19^ where it plays protective roles in the intestinal barrier, stimulates the production of SCFAs and has positive immunomodulatory effects.^20^ However, *Bifidobacterium* levels are often decreased under conditions of liver injury, which may be related to the pathophysiology of various diseases. In this context, liver status in patients with SLE and metabolic disorders is critical to understanding the interactions between these conditions.^21^ The liver‐gut axis, which represents the bidirectional communication between the liver and gastrointestinal tract, plays a key role in the pathophysiology of several systemic diseases, including SLE and low‐grade MI.^22^, ^23^, ^24^ Investigating these interactions may reveal new therapeutic targets and interventions to modulate the gut microbiota, restore gut barrier integrity and reduce liver inflammation. This approach is essential to address gut dysbiosis and inflammatory activity, which are central to the pathogenesis of SLE.^25^, ^26^ Holistic interventions combining dietary adjustments, microbiota‐targeted therapies and weight management show promise in the field of precision nutrition, potentially improving clinical outcomes and overall patient well‐being. Therefore, the analysis of the intestinal microbiota has the potential to improve both prevention and treatment of inflammatory diseases, based on a detailed understanding of physiopathological phenotypes.^26^, ^27^

Our hypothesis was that the differences in liver status health in both inflammatory conditions may be partly explained by distinctive gut microbiota composition. Thus, the aim of this research was to compare common and unshared anthropometric, biochemical, and inflammatory markers in patients with different types of inflammation (low‐grade MI as reference and SLE), as well as to investigate the relationships between liver markers and gut microbiota to highlight potential associations between type of inflammatory condition, hepatic variables, and microbiota composition.

## 2 MATERIALS AND METHODS

### 2.1 Study design

This investigation is part of the ‘METAINFLAMMATION’ project (ref. Y2020/BIO‐6600), which is a prospective and controlled study. The recruitment of the participants took place from January 2022 to June 2023 at the Internal Medicine Service of Puerta de Hierro Majadahonda University Hospital in Madrid, Spain. Recruitment procedures involved participant acceptance into the study and the completion of informed consent documents. The trial adhered to the principles of the Declaration of Helsinki and obtained approval from the Research Ethics Committee of Puerta de Hierro Majadahonda University Hospital (file number PI 164‐21). All data collection procedures were executed in strict accordance with approved ethical guidelines and hospital protocols.

### 2.2 Participants

This research involved 60 adults sequenced upon arrival, older than 18 years old, both men and women, of Caucasian and Hispanic ancestry. The participants were assigned into two groups according to the medical diagnoses received: MI and SLE, both diseases are considered complex conditions that are characterized by an inflammatory state.^28^ The MI group included patients presenting a combination of obesity and metabolic syndrome components, considered to be manifestations of low‐grade MI,^29^ who were used as a standard as well as the hospital reference values. The diagnostic criteria established by the World Health Organization (WHO) and the National Education Program on Cholesterol (NECP) were used to identify patients with obesity and metabolic syndrome as control,^30^ while for the SLE group, the classification criteria established by the European League Against Rheumatism/American College of Rheumatology were applied.^31^

### 2.3 Inclusion criteria

The participants met the following inclusion criteria: age > 18 years, a body mass index (BMI) > 18 and < 50 kg/m^2^ and diagnosis of SLE and MI confirmed by the medical staff of the Internal Medicine service of the Puerta de Hierro Majadahonda University Hospital (Madrid, Spain). Patients with obesity and metabolic syndrome presented a series of alterations such as excessive adiposity, glucose intolerance, central obesity, dyslipidemia, and hypertension.^32^ Meanwhile, patients diagnosed with SLE in a stable state and under supervised medical treatment were selected to reduce possible biases. Various clinical parameters were evaluated in these patients, such as serological activity (SA), the presence of active disease (AD), the achievement of complete remission (CR) and the maintenance of a low disease activity state (LDAS). Additionally, anti‐dsDNA antibodies and different treatment regimens received were considered for adjustments.^33^, ^34^, ^35^ SLE activity was assessed using the SLE Disease Activity Index (SLEDAI‐2 k), while organ damage was assessed using the SLICC/American College of Rheumatology (ACR).^34^, ^36^ In addition, only sequenced patients who provided faecal samples adequately were considered for this investigation.

### 2.4 Exclusion criteria

Exclusion criteria included the presence of severe psychiatric disorders, the current use of body weight‐modifying agents, difficulty for scheduling appointments, pregnancy, lactation, and patients with changes in pharmacological prescription 1 year before. The consumption of probiotics, antibiotics or supplements proved to alter the gut microbiota composition at least 3 weeks before the collection of the faecal samples was considered as exclusion criteria.

### 2.5 Anthropometrics and clinical measurements

Anthropometric measurements were assessed by a skilled dietitian using validated techniques.^37^ Body weight was determined using a bioimpedance scale (TANITA SC‐330; Tanita Corporation Pais), which also provided estimates of body composition (skeletal muscle mass, visceral fat, estimated metabolic age and estimated basal metabolic rate). Waist circumference was measured with a standard tape measure following established protocols and performed by trained dietitians. BMI was calculated as the ratio of body weight to the square of height (kg/m^2^).^37^ Systolic and diastolic blood pressures were measured with a sphygmomanometer, following standardized criteria based on international guidelines.^38^ Mediterranean diet adherence was assessed using a validated questionnaire of 14 points.^39^, ^40^

### 2.6 Biochemical data

Blood samples were assessed under fasting conditions through venipuncture. The samples underwent analysis for leukocytes, lymphocytes, neutrophils, monocytes, mean corpuscular volume, platelets, erythrocyte sedimentation rate (ESR) and erythrocyte distribution width (RDW) utilizing an SYSMEX XN‐20 automated haematology analyser (Roche, Basel, Switzerland) following validated procedures. The neutrophil/lymphocyte ratio was calculated directly from the measured values.^37^ Routine biochemical markers, including glucose, total cholesterol, glycated haemoglobin, urea, gamma glutamyl transpeptidase (GGT), high density lipoprotein (HDL), low density lipoprotein (LDL), triglycerides, alanine aminotransferase (ALT) and aspartate aminotransferase (AST) were measured following standardized hospital protocols using a quality‐controlled autoanalyser (Atellica™ Solution Pais) as per established criteria.^41^ C reactive protein (CRP), fibrinogen, insulin, N‐terminal pro‐brain natriuretic peptide type B (NT ProBNP), IL‐6, and prothrombin activity also followed standardized procedures, primarily employing ELISA kits (Sigma‐Aldrich ELISA Kit Pais) as outlined by the suppliers. The Homeostatic Model Assessment for Insulin Resistance (HOMA‐IR index) estimates insulin resistance in the body, which was calculated as fasting glucose (mg/dL) × .0555 × fasting insulin (μUI/mL)/22.5.^42^ Triglycerides‐glucose index (TyG) was used as an indicator of insulin resistance and the risk of developing metabolic diseases such as type 2 diabetes and cardiovascular diseases and was computed following the validated formula: (ln [TG (mg/ dL) × FPG (mg/dL)/2].^43^ The AST/ALT ratio was calculated directly from the measured values. Fatty liver index (FLI) estimates the probability of having nonalcoholic fatty liver disease (NAFLD) in adults. This score was calculated as: FLI = (e .953×log_e_ (triglycerides) + .139×BMI + .718×log_e_ (GGT) + .053×(waist circumference−15.745)/(1 + e .953×log_e_ (triglycerides) + .139×BMI + .718×log_e_ (GGT) + .053×(waist circumference−15.745) × 100.^44^ Hepatic steatosis index (HSI) is a tool that helps estimate the severity of non‐alcoholic fatty liver by evaluating certain clinical and laboratory parameters. HSI was calculated as: HSI = 8 × (ALT/AST ratio) + BMI (+2, if female; +2, if diabetes mellitus).^45^ A lipid accumulation product (LAP) is an index calculated by waist circumference and triglyceride, which reflects lipid toxicity.^46^

### 2.7 Metagenomic analysis

Faecal samples were collected using OMNIgene® •GUT kits (DNA Genotek, Ottawa, ON, Canada), according to the supplier instructions.^47^ Bacterial DNA was isolated with the QIAamp® DNA kit (Qiagen, Hilden, Germany) following the manufacturer's protocol and the V3‐V4 hypervariable regions of the 16 S rRNA gene were amplified by paired‐end DNA sequencing in the MiSeq System (Illumina, San Diego, CA, USA) at Novogene Sequencing‐ Europe Service (Cambridge, United Kingdom). Also, the primers used for the PCR reactions were (16S Amplicon PCR Forward Primer =5 0 TCGTCGGCAGCGTCAGATGTGTATAAGAGACAGCCTACGGGNGGCWGCAG; 16S Amplicon PCR Reverse Primer = 5 0 GTCTCGTGGGCTCGGAGATGTGTATAAGAGACAGGACTACHVGGGTATCT AATCC). PCR reactions were carried out with 15 μL of Phusion® High—Fidelity PCR Master Mix (New England Biolabs); .2 μM of forward and reverse primers, and about 10 ng template DNA. Thermal cycling consisted of initial denaturation at 98°C for 1 min, followed by 30 cycles of denaturation at 98°C for 10 s, annealing at 50°C for 30 s, and elongation at 72°C for 30 s and 72°C for 5 min. The PCR products were purified using magnetic beads and the samples were mixed in equidensity ratios based on the concentration of PCR products. After thorough mixing, the PCR products were detected, and target bands were recovered. For library preparation, sequencing libraries were generated, and indexes were added. The library was checked with Qubit and real‐time PCR for quantification and bioanalyzer for size distribution detection. Quantified libraries were pooled and sequenced on Illumina platforms, according to effective library concentration and data amount required. For bioinformatic analysis, paired‐ end reads were assigned to samples based on their unique barcode and truncated by cutting off the barcode and primer sequence. Paired‐end reads were merged using FLASH (V1.2.7, http://ccb.jhu.edu/software/FLASH/),^48^ while quality filtering on the raw tags were performed using the FASTP (version 0 .23. 1) software to obtain high‐quality Clean Tags.^49^ The tags were compared with the reference database (Silva database (16S/ 18S), https://www.arb‐silva.de/; Unite Database using the search (https://github.com/torognes/vsearch/) to detect chimera sequences, and then the chimera sequences were removed.^50^ For the Effective Tags obtained previously, denoise was performed with DADA2 or deblur module in the QIIME2 software (Version QIIME2‐202202) to obtain initial ASVs (Amplicon Sequence Variants). Species annotation was performed using QIIME2 software (SILVA Database) and to study phylogenetic relationship of each ASV and the differences of the dominant species among different samples(groups), multiple sequence alignment was performed using QIIME2 software and displayed with R software (Version 2, vegan package).

### 2.8 Statistical analyses

Variables were expressed as means ($\bar{x}$) and standard deviations (SD) for quantitative variables and number of cases (*n*) and proportions (%) for qualitative variables. Normality of the data was assessed by Shapiro–Wilk test. Student's *t* and Mann–Whitney tests were implemented depending on normality to compare the means of the continuous variables at the beginning of the study and the categorical variables were statistically screened using the chi‐square (*χ* ^2^) test. The analysis of microbiota was evaluated comparing the two types of inflammatory diseases, adjusted by BMI, age and sex to avoid potential confounders influences. Alpha diversity (related to the distribution of species abundances in a sample) profiling between types of inflammatory diseases was calculated by determining the Shannon index at genus level, which are considered a measure commonly used in to compare diversity between different samples or communities using MicrobiomeAnalyst (https://www.microbiomeanalyst.ca/),^51^ compared by Mann–Whitney test and visualized using boxplot. Beta diversity (which assesses the similarity between microbial communities) was calculated using Bray Curtis index and PERMANOVA test and then visualized by means of principal coordinate analysis (PCoA). In addition, linear discriminant analysis (LDA) effect size (LEfSe) (http://huttenhower.sph.harvard.edu/galaxy/) was used to compare groups (with log LDA score = 2.0 and FDR adjustment) and visualize the results using taxonomic bar charts. Zero‐inflated Gaussian (MetagenomeSeq) analysis was for finding families that differed significantly in abundance between normal body weight and obese subjects (using FDR adjustment for the significant results). To generate the volcano plot comparing bacterial abundance between two disease groups, the log_2_ fold change and *p*‐values were calculated using R. Potential interactions between bacteria, type of disease and liver's status were investigated with general linear regression models that introduced the corresponding interaction terms into the models, these models were adjusted for age, sex and adherence to Mediterranean diet in order to avoid possible bias, using Stata 12 (StataCorp LLC, College Station, TX, USA; http://www.stata.com). Mediation analysis was assessed using structural equation modelling approach.^49^ The normalization of microbiota data was performed according to the most appropriate statistical analysis method, considering centered‐log ratio for regression models.^52^, ^53^ A *p* value of *p* < .05 was considered statistically significant.

## 3 RESULTS

### 3.1 Assessment of anthropometric, body composition and clinical markers in patients with low‐grade metabolic inflammation and systemic lupus erythematosus

Table 1 shows the comparisons of anthropometric, body composition and biochemical variables between subjects with low‐grade metabolic inflammation (MI) and systemic lupus erythematosus (SLE). Regarding anthropometric measurements, individuals with low‐grade MI exhibited higher values for body weight (*p* < .01), waist circumference (*p* < .01), skeletal muscle mass (*p* < .001), visceral fat (*p* < .001) and estimated basal metabolic rate (*p* < .001), compared with individuals with SLE. A marginal trend was observed in BMI (*p* < .07) and no significant differences were found in systolic and diastolic blood pressure (*p* > .05) between the two groups. No significant differences were found for the adherence to Mediterranean diet (*p* = .77).

**TABLE 1:** Comparison of anthropometric measurements, body composition, biochemical and clinical markers between two types of inflammatory conditions (low‐grade metabolic inflammation and systemic lupus erythematosus) in the METAINFLAMMATION cohort.

	Hospital reference values (28)^a^	Low‐grade metabolic inflammation (MI)	Systemic lupus erythematosus (SLE)	*p* value^*^
Variables		*n* = 33	*n* = 27	
Age (years)	NA	60 (11)	52 (14)	**.03**
Gender = Woman (%)	NA	15 (45.5)	25 (92.6)	**<.001**
Body weight (Kg)	NA	87.8 (16.2)	74.2 (17.4)	**<.01**
Body mass index (Kg/m^2^)	18.5–24.9	31.1 (3.8)	28.8 (5.6)	.07
Waist circumference (cm)	Male <94 Female <80	109.4 (9.9)	97.5 (14.4)	**<.01**
Skeletal muscle mass (Kg)	NA	53.7 (10.8)	43.6 (7.3)	**<.001**
Visceral fat	NA	14.1 (4.4)	9.2 (4.4)	**<.001**
Estimated metabolic age (years)	NA	67 (12)	56 (20)	**.02**
Estimated basal metabolic rate (Kcal)	NA	1695 (333)	1400 (232)	**<.001**
Glucose (mg/dL)	60–100	106.6 (18.2)	91.5 (13.4)	**<.001**
Glycated haemoglobin (%)	4.5–6.4	5.8 (.7)	5.5 (.4)	**.03**
Insulin (μUI/mL)	0–29.1	13.0 (9.8)	11.3 (10.4)	.20
HOMA‐IR	.5–1.9	2.9 (2.8)	2.4 (2.7)	.23
Total cholesterol (mg/dL)	150–200	178.8 (35.1)	167.4 (32.2)	.05
HDL‐cholesterol (mg/dL)	45–90	51.4 (20.1)	56.1 (12.2)	.06
LDL‐cholesterol (U/L)	70–160	102.9 (31.1)	92.5 (28.1)	.18
Triglycerides (mg/dL)	30–200	135.5 (47.6)	105.5 (85.4)	**<.01**
TyG index	< 2	4.8 (.2)	4.5 (.3)	**<.01**
Urea (mg/dL)	7–20	39.1 (10.8)	39.7 (18.8)	.60
ESR (mm)	21–50	9.2 (8.4)	15.4 (13.5)	.06
Systolic blood pressure (mmHg)	< 120	141.2 (19.9)	131.5 (20.4)	.07
Diastolic blood pressure (mmHg)	< 80	81.5 (12.8)	76.0 (13.9)	.12
Mediterranean diet adherence	> 7	6.5 (1.9)	6.7 (2.0)	.77

*Note*: Data presented as mean ($\bar{x}$), standard deviation (SD), and *p* values. *p* value refers to the comparison of variables' mean between patients with MI and SLE using *t*‐test or Mann–Whitney test, according to the distribution of the data assessed by Shapiro–Wilk test.

Abbreviations: ESR, erythrocyte sedimentation rate; HDL, high density lipoprotein; HOMA‐IR, Homeostatic Model Assessment for Insulin Resistance; LDL, low density lipoprotein; TyG Index, triglyceride‐glucose index.

^a^ Laboratory's values of reference for each variable are shown in the second column (NA: not applicable).

* Bold indicates significance level at *p* value < .05.

In addition, the biochemical comparison between participants with low‐grade MI and those with SLE revealed remarkable differences. Patients with MI had higher glucose levels (*p* < .001), and elevated triglycerides (*p* < .01) compared to those with SLE (Table 1).

Specifically, the HOMA‐IR and TyG index, showed distinctions between the two inflammatory conditions. While the mean HOMA‐IR indicates a higher but non‐significant value (*p* = .23) in MI patients compared to SLE, the TyG index exhibits a statistically significant elevation in MI individuals (*p* < .01), suggesting a higher insulin resistance state evaluated by this index. Nonetheless, LDL levels did not demonstrate a significant difference between the groups (*p* = .18).

### 3.2 Hepatic and inflammatory profile of patients with low‐grade metabolic inflammation and systemic lupus erythematosus of METAINFLAMMATION cohort

A comparison of several liver markers was performed between individuals with low‐grade MI and SLE within the METAINFLAMMATION cohort (Table 2). The levels of AST showed a trend towards significance (*p* = .06), with slightly higher values observed in the low‐grade MI group compared with SLE. Conversely, ALT levels were significantly elevated in the low‐grade MI group compared with SLE (*p* < .01), while the AST/ALT ratio was significantly lower (*p* < .001). Furthermore, GGT levels were also significantly higher in the low‐grade MI group compared with SLE (*p* < .01). Likewise, the HSI, FLI, and LAP indexes were all significantly higher in the low‐grade MI group (*p* < .05).

**TABLE 2:** Comparison of liver markers among the two types of inflammatory conditions (low‐grade metabolic inflammation and systemic lupus erythematosus) in the METAINFLAMMATION cohort.

	Hospital reference values (28)^a^	Low‐grade metabolic inflammation (MI)	Systemic lupus erythematosus (SLE)	*p* value^*^
Variables		*n* = 33	*n* = 27	
AST (U/L)	6–40	25.1 (7.1)	21.9 (4.5)	.06
ALT (U/L)	6–40	32.1 (11.2)	22.0 (13.3)	**<.01**
GGT (U/L)	10–50	31.8 (18.3)	18.6 (11.9)	**<.01**
AST/ALT ratio	.5–2	.8 (.2)	1.2 (.4)	**<.001**
HSI	<36	42.8 (5.4)	38.7 (7.7)	**.02**
FLI	< 30	74.2 (18.7)	45.9 (33.3)	**<.01**
LAP	NA	75.2 (35.1)	47.8 (36.6)	**<.01**

*Note*: Data presented as mean ($\bar{x}$), standard deviation (SD), and *p* values. *p* value refers to the comparison of variables' mean between patients with MI and patients with SLE using *t*‐test or Mann–Whitney test, according to the distribution of the data assessed by Shapiro–Wilk test.

Abbreviations: ALT, alanine transaminase; AST, aspartate aminotransferase; FLI, fatty liver index; GGT, gamma glutamyl transferase; HSI, Hepatic Steatosis Index; LAP, lipid accumulation product.

^a^ Laboratory's values of reference for each variable are shown in the second column (NA: not applicable).

* Bold indicates significance level at *p* value < .05.

The comparison of biochemical and haematological variables between participants with low‐grade MI and those with SLE are shown in Table 3. No significant differences were found between the groups, suggesting comparable levels of systemic inflammation evaluated by these clinical markers.

**TABLE 3:** Comparison of haematological, inflammatory and coagulation markers among the two types of inflammatory conditions (low‐grade metabolic inflammation and systemic lupus erythematosus) in the METAINFLAMMATION cohort.

	Hospital reference values (28)^a^	Low‐grade metabolic inflammation (MI)	Systemic lupus erythematosus (SLE)	*p* value^*^
Variables		*n* = 33	*n* = 27	
Leukocytes (10E^3^/μL)	4–11.5	6.5 (1.7)	6.0 (2.5)	.12
Neutrophils/lymphocytes ratio	1–7	2.3 (.6)	3.1 (4.8)	.58
Platelets (10E^3^/μL)	150–400	235.5 (51.5)	244.3 (79.8)	.86
NT proBNP (pg/mL)	37–125	60.8 (60.0)	74.4 (55.5)	.14
aPTT (s)	23–36	29.8 (2.7)	34.4 (9.5)	.08
RDW (%)	8–14.8	13.7 (.7)	14.2 (1.4)	.21
Fibrinogen (mg/dL)	200–400	383.3 (91.8)	388.9 (130.3)	.89
C reactive protein (mg/L)	.1–10	3.5 (3.8)	4.8 (6.3)	.68
IL‐6 (pg/mL)	0–4.4	3.4 (1.8)	3.6 (1.7)	.26

*Note*: Data are presented as mean ($\bar{x}$), standard deviation (SD), and *p* values. *p* value refers to the comparison of variables' mean between patients with SLE and patients with MI using *t*‐test or Mann–Whitney test, according to the distribution of the data assessed by Shapiro–Wilk test.

Abbreviations: aPTT, Activated Partial Thromboplastin Time; IL‐6, Interleukin‐6; NT proBNP, Natriuretic Peptide Tests; RDW, Red Cell Blood Distribution Width.

^a^ Laboratory's values of reference for each variable are shown in the second column (NA: not applicable).

* The significance threshold was set at *p* < .05.

### 3.3 Richness and diversity of gut microbiota in SLE and low‐grade MI patients

**FIGURE 1:**
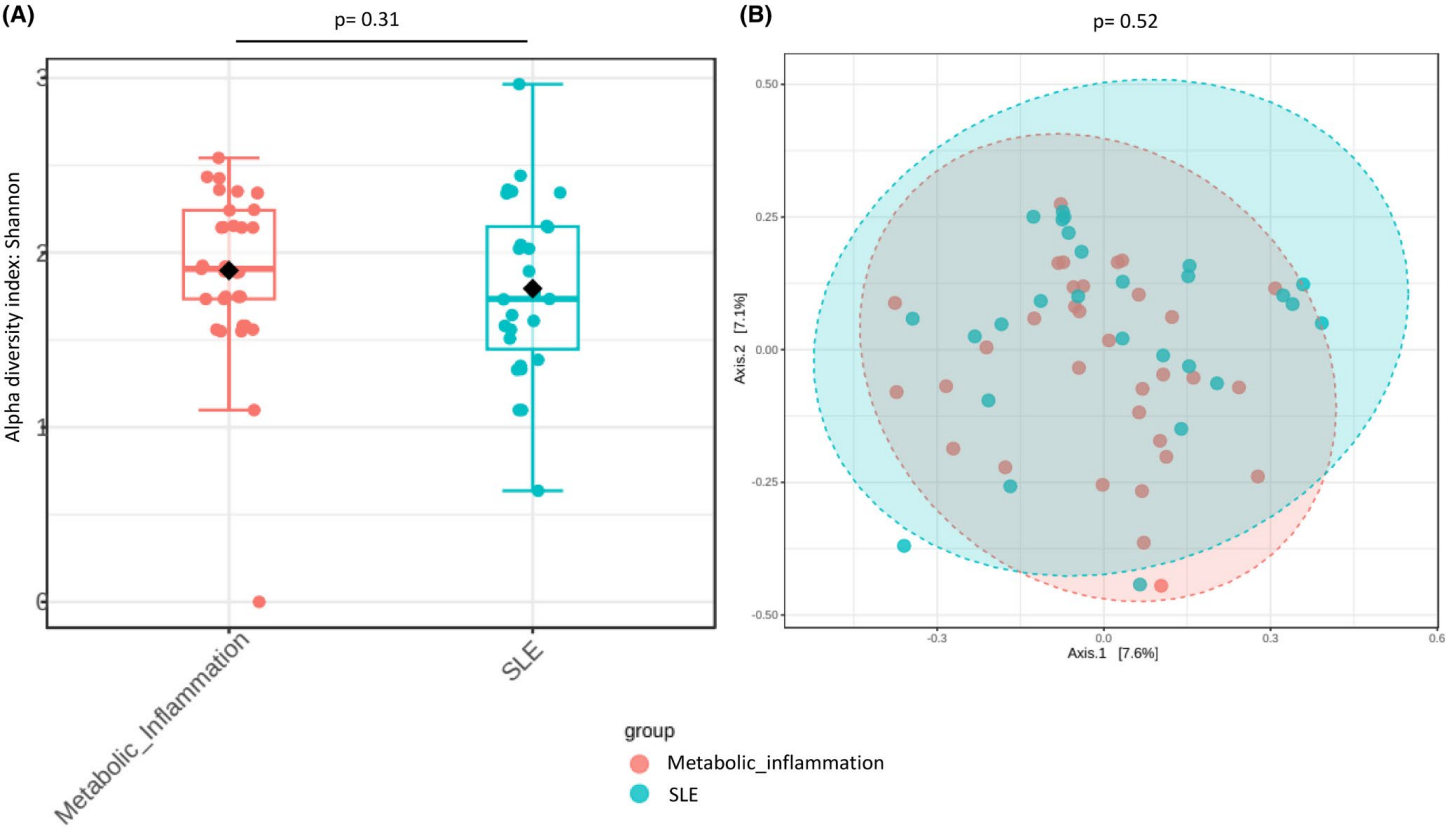
(A) Alpha diversity analysis evaluated by Shannon index, according to the type of disease. Orange boxes represent low‐grade MI patients and blue boxes represent patients with SLE. (B) Principal coordinate analysis for beta diversity calculated using Bray Curtis index and PERMANOVA test. Red circles represent low‐grade MI participants and blue circles represent SLE participants.

The analysis of gut microbiota richness between participants with low‐grade MI and participants with SLE showed no significant differences (*p* = .37). Likewise, the analysis of alpha diversity showed no relevant differences when evaluated by Shannon index (*p* = .31) (Figure 1A). Similarly, no significant differences in beta diversity were found using Bray Curtis distances (*p* = .52) (Figure 1B).

### 3.4 Gut microbiota composition and structure in SLE and low‐grade MI participants

**FIGURE 2:**
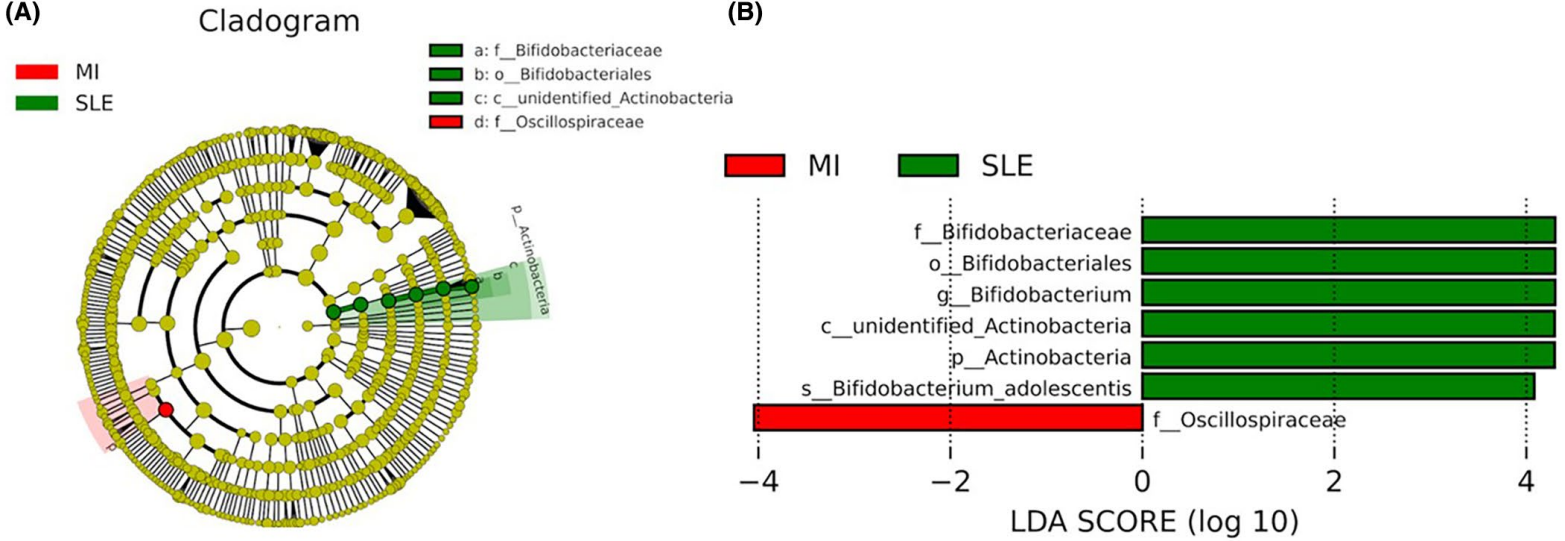
Differently abundant taxa in low‐grade MI patients and SLE patients, based on LEfSe analysis. The colours represent the group in which the indicated taxa are more abundant compared to the other group. In a taxonomic cladogram, each successive circle represents a different phylogenetic level. (A) Cladogram: The order from the center to the outside is phylum, class, family, and genus levels. Different taxa are listed on the right side of the cladogram. (B) Linear discriminant analysis. The most differentially abundant taxa between sexes are represented in a bar graph according to the LDA score (log 10), an estimation of the effect size. Only taxa meeting a *p* < .05 and LDA score significant threshold | > 2| are shown. Red, bacterial taxa statistically overrepresented in low‐grade MI participants; green, bacterial taxa overrepresented in participants with SLE.

A comparative visualization of the taxonomic structure of the gut microbiota between these two types of inflammatory disease was performed to provide a detailed scope and to identify important patterns, differences, and relationships among the analysed groups. For this purpose, LEfSe analysis was used to evaluate microbiota differences between individuals with SLE and MI (Figure 2).

The linear discriminant analysis effect size showed a preponderance of families from the Oscillospiraceae family in participants with low‐grade MI. By contrast, microorganisms from the Actinobacteria phylum, such as Bifidobacteriales order, Bifidobacteriaceae family, *Bifidobacterium* genus and *Bifidobacterium adolescentis* species were overrepresented in SLE participants, evaluated by LEfSe analysis.

**FIGURE 3:**
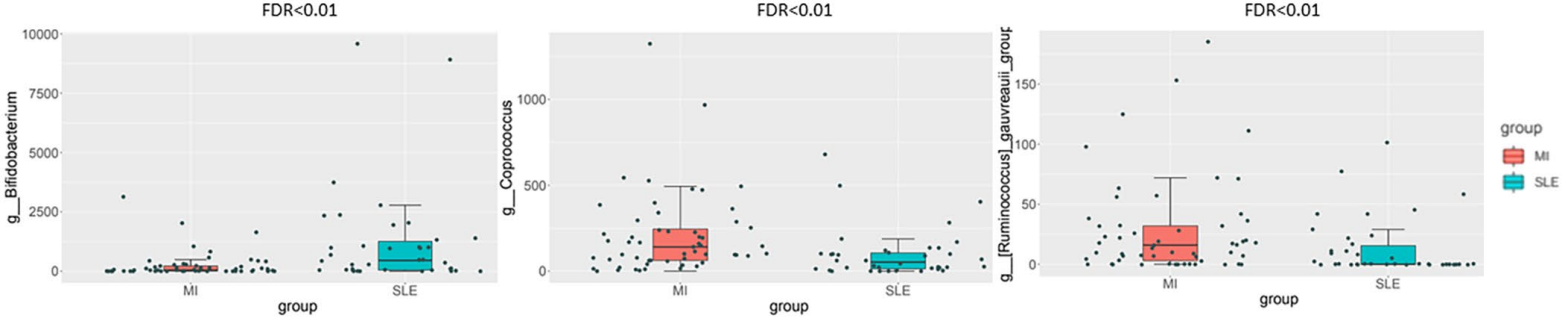
Box plots comparing bacterial abundance by MetagenomeSeq analysis between two types of inflammatory conditions: Low‐grade MI (red box) and SLE (blue box). FDR <.01: False discovery rate adjusted *p* value. FDR <.01. FDR <.01. FDR <.01.

Moreover, MetagenomeSeq analysis was performed to identify bacterial families significantly different between the two types of inflammatory conditions. Figure 3 show that *Bifidobacterium* (FDR <.01), *Rumminococus* (FDR <.01), and *Coprococcus* (FDR <.01) genera presented a significant differential abundance depends on the disease (Figure 3), being *Bifidobacterium* genus significantly increased in SLE in comparison with low‐grade MI.

Similarly, Figure S1 showed a volcano performed to represent both the magnitude and statistical significance of differences between SLE and low‐grade MI. The volcano plot shows the significantly up‐regulated difference between bacteria and significantly down‐regulated difference between groups of disease. This analysis also showed that *Bifidobacterium* genus was upregulated in SLE participants in contrast with low‐grade MI (supplementary material).

### 3.5 Assessment of the relationship between the inflammatory condition, gut microbiota, and liver markers

**FIGURE 4:**
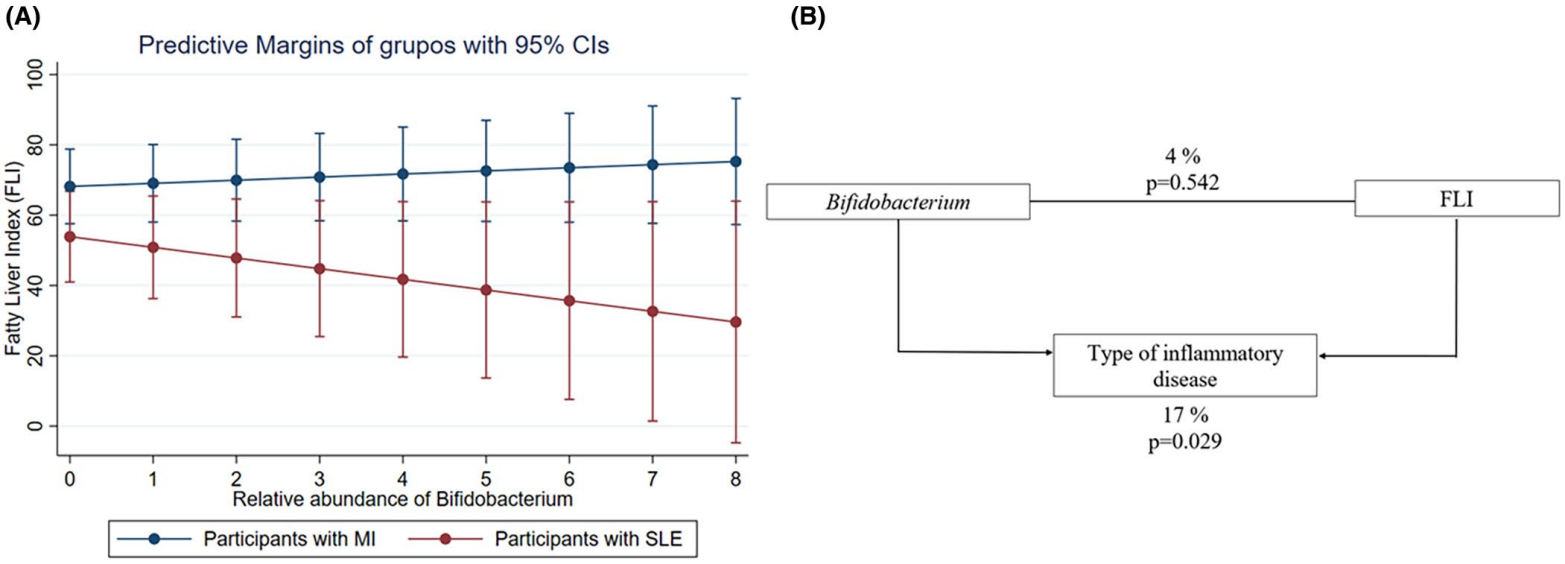
(A) Predicted values of Fatty Liver Index (FLI) in patients with low‐grade metabolic inflammation (MI) and Systemic Lupus Erythematosus (SLE), according to the relative abundance of *Bifidobacterium* genus in the gut (*R* ^2^ = .24; *p* = .02). Blue line represents values for patients with low‐grade MI and the red line represents values for SLE. The model was adjusted for age, sex, and adherence to Mediterranean diet to avoid bias. (B) Type of inflammatory disease (low‐grade MI or SLE) mediated relationship between *Bifidobacterium* and FLI.

A regression analysis was performed to analyse the potential interaction between liver status measured by FLI index (as dependent variable) *Bifidobacterium* abundance levels (as independent variable) and type of inflammatory disease (low‐grade MI and SLE). A significant interaction emerged between these variables, as represented in Figure 4A (*R* ^2^ = .24; *p* = .02), showing that participants with SLE (red line in Figure 4A) and with higher abundance of *Bifidobacterium* genus presented lower predicted values of FLI. However, participants with low‐grade MI (blue line in Figure 4A) presented the same values of FLI, regardless of whether the abundance of *Bifidobacterium* was high or low. In addition, the mediation analysis assessed the relationship between the type of inflammatory disease, *Bifidobacterium* and FLI, using a mediation equation (Figure 4B). These findings suggest that the association between *Bifidobacterium* abundance levels and FLI varies depending on the type of inflammatory condition, showing an effect modification depending on the type of disease. Indeed, for SLE participants, higher abundance of the genus *Bifidobacterium* was associated with low FLI values (Figure 4A). Furthermore, this relationship still lasted after controlling for the effect of the mediating variable (Figure 4B).

## 4 DISCUSSION

In this research, patients with SLE and those with low‐grade MI were compared to analyse differences and similarities in anthropometric, biochemical, inflammatory and metagenomic variables. The results showed that both conditions present different inflammatory profiles, but with shared characteristics. An imbalance in gut microbiota was found to contribute to chronic inflammation and autoimmunity,^54^, ^55^ highlighting the importance of gut microbiota in the pathophysiology of both SLE and low‐ grade MI.^56^, ^57^ This study suggests that understanding phenotypic differences and the impact of the microbiota on liver health is key to improving prevention and treatment strategies in these inflammatory diseases.

In this investigation, significantly higher values in anthropometric measurements and body composition were observed in the group with low‐grade MI, with parameters such as body weight, waist circumference, total muscle mass, visceral fat and metabolic age being the most important. These factors are predictive indicators of metabolic disorders. The results revealed important physiological differences between patients with low‐grade MI and those with SLE, supporting the relationship between alterations in anthropometric measurements and body composition in individuals with low‐grade MI.^58^, ^59^ These findings are consistent with previous studies that have also demonstrated an association between increased anthropometric parameters, such as body weight and waist circumference, and the development of metabolic complications.^60^ Also, comparative analysis of biochemical markers revealed significantly higher levels of glucose and glycosylated haemoglobin in patients with low‐grade MI, suggesting higher dysglycaemia and poorer long‐term glycemic control. In addition, this group showed higher triglyceride levels and a tendency towards increased total cholesterol, typical features of dyslipidemia associated with metabolic disorders.^61^, ^62^ Concomitantly, elevated values of ALT, GGT, HSI, FLI, LAP and TyG index were observed, reinforcing the relationship between low‐grade MI and liver damage, showing a higher prevalence of hepatic steatosis and lipid accumulation.^63^ These findings are consistent with the pathophysiology of metabolic disorders associated with low‐grade inflammation, where excess liver fat contributes to liver dysfunction and metabolic complications.^61^ Previous studies have demonstrated the association between hepatic steatosis, low‐grade MI and metabolic dysfunction, highlighting the link with excess body weight and inflammatory processes related to hepatic fat accumulation.^64^, ^65^, ^66^

In the analysis of haematological, inflammatory and coagulation markers, no significant differences were found between groups, suggesting no differences in the inflammatory markers evaluated in this research for this population. Indeed, inflammatory markers are triggered by different stimulus and injuries. Interestingly this pathophysiological mechanism may have an impact on microbiota abundance, while microbiota also can produce inflammatory molecules such as lipopolysaccharides (LPS) that can interact with few processes related to SLE and MI. The lack of statistical significance in CRP levels between groups with chronic inflammation, such as SLE and low‐grade MI, may be due to several reasons. Although both share a chronic inflammatory state, the underlying mechanisms are different: in SLE, the inflammation is of autoimmune origin, whereas in MI it is related to obesity and insulin resistance, which may affect CRP expression.^10^ In addition, CRP is a non‐specific marker of systemic inflammation and therefore may not accurately reflect inflammatory processes in these conditions.^67^ Factors such as genetics, age, diet, gender and the use of medications, such as immunosuppressants in SLE,^68^ may also influence CRP levels and lead to high variability between individuals.^69^ Since CRP is a marker of acute inflammation, it may not be the best indicator to assess chronic low‐grade inflammatory processes,^70^ with other biomarkers, such as interleukins or TNF‐α, being more sensitive.^71^ Finally, aspects such as sample size and variability between groups may have contributed to the lack of statistical significance, but the values in both cases are over the media of the hospital records. However, no differences between them were found, which may have explained by endogenous and external factors.

Regarding microbiota analysis, no significant differences were found in richness and diversity when comparing the two types of conditions. Previous studies have documented that the diversity of gut microbiota tends to decrease in chronic inflammatory diseases, such as inflammatory bowel disease, indicating a possible impact of the chronic state of the host.^72^ However, disparities in taxonomic structure between the two conditions studied were observed in this research. Specifically, participants with low‐grade MI showed a predominance of the Oscillospiraceae family, according to LEfSe analysis. This family has previously been linked to obesity, metabolic syndrome and other metabolic disorders.^73^ Previous studies have suggested that elevated levels of Oscillospiraceae are associated with insulin resistance, a hallmark of metabolic syndrome, and may be implicated in chronic low‐grade inflammation and metabolic dysfunction.^73^ On the other hand, patients with SLE showed significantly higher abundance of the *Bifidobacterium* genus compared to those with low‐grade inflammatory condition (MI group). *Bifidobacterium*, a common bacterial genus in the human gastrointestinal tract, has been associated with symptom attenuation in SLE patients and modulation of liver function.^74^ Previous studies have found an increased presence of *Bifidobacterium* in faecal samples from patients with SLE in remission compared to those with active disease, suggesting a possible protective role of this bacterial genus in the progression of SLE.^14^, ^75^, ^76^

The multiple regression analysis indicated that *Bifidobacterium* abundance differentially impacts the FLI according to inflammatory disease type, suggesting a possible protective effect of this bacterial genus in patients with SLE, in contrast to those with low‐grade MI. FLI widely used to detect hepatic steatosis,^77^ is associated with systemic inflammation and general health status.^78^ Previous studies have pointed to the probiotic potential of *Bifidobacterium* in liver disease, Previous studies have pointed to the probiotic potential of *Bifidobacterium* in liver disease, with benefits in restoring gut microbiota and liver function, as well as reducing inflammation with benefits in restoring gut microbiota and liver function, as well as reducing inflammation.^79^ Liver disorders affect the composition of the gut microbiota, leading to dysbiosis and gut barrier dysfunction, which aggravates liver inflammation, especially in patients with SLE.^80^ Although an increased presence of *Bifidobacterium* has been observed in patients with SLE compared to those with metabolic inflammation, its specific role in SLE is not fully understood, requiring further research. However, studies in NAFLD indicate that *Bifidobacterium* may reduce hepatic fat accumulation, inflammation and intestinal permeability, suggesting a therapeutic potential in liver disorders.^81^, ^82^ For example, one study showed that supplementation with *Bifidobacterium adolescentis* improved visceral fat accumulation and increased insulin sensitivity in a model of metabolic syndrome.^83^ Other research found that administration of *Bifidobacterium* and *Lactobacillus* strains modulated the severity of experimental autoimmune hepatitis in mouse models.^84^ In addition, supplementation with *Bifidobacterium pseudocatenulatum* CECT 7765 has been reported to improve inflammatory status in obese insulin‐resistant children.^85^ These findings point to a protective effect of *Bifidobacterium* on liver health, although the results need to be interpreted with caution due to the limited evidence in this area. In this context, the need for the personalization of therapies is presented as a crucial element to effectively manage different types of inflammatory diseases, considering the complex interaction between the composition of intestinal microbiota, liver health and type of inflammation, where also genetics, gut derived metabolites or epigenetic marks may have a mediating role.^86^, ^87^, ^88^

This research has several notable strengths. First, a low‐grade inflammation and an autoimmune‐related inflammation were analysed and compared, providing a robust evaluation. Furthermore, the inclusion of a wide range of anthropometric, biochemical, inflammatory, and hepatic markers, together with the composition of the gut microbiota, provided a comprehensive and comparative perspective of this population, with possible implications for nutrition and precision medicine. However, the study has limitations in terms of the sample size in both groups, which is relatively small.

In conclusion, this investigation comprehensively compared anthropometric, body composition, biochemical and clinical markers between individuals with low‐grade MI and exacerbated chronic inflammation (SLE), showing distinct profiles in both conditions concerning several inflammatory markers. Patients with low‐grade MI had higher values for anthropometric and adverse body composition measurements, together with less favourable biochemical and liver profiles as compared to SLE. Differences in gut microbiota were also observed, with a higher abundance of *Bifidobacterium* in SLE patients. Analyses suggest that gut microbiota, particularly *Bifidobacterium* abundance, differentially influences each condition, showing a significant interaction with the FLI as marker of liver status. These results underline the importance of considering gut microbiota in the precision personalized management of inflammatory diseases, as a higher abundance of *Bifidobacterium* could have a positive impact on liver health in SLE patients, putatively mediated by inflammatory processes.

### 4.1 Perspectives and significance

This research contributes to a deeper understanding of the complex interactions between different types of inflammation diseases, gut microbiota, and hepatic health, with implications for the development of novel therapeutic interventions and clinical management strategies in inflammatory conditions. Understanding these differences and the role of the gut microbiota opens new possibilities for personalized therapeutic interventions in the management of inflammatory diseases. By recognizing the importance of gut microbiota composition in disease pathogenesis, clinicians can tailor treatment strategies to target dysbiosis and promote a healthy gut environment, ultimately improving patient outcomes.

## AUTHOR CONTRIBUTIONS

Conceived and designed research (AC‐S, JAM), performed investigations and experiments (LCH‐S, AC‐S, MM‐U, AH‐G, SM‐P), analysed data (LCH‐S, AC‐S, MM‐U, AH‐G), interpreted results of experiments (LCH‐S, AC‐S, JAM), prepared figures (LCH‐S, AH‐G, AC‐S), drafted manuscript (LCH‐S, AC‐S), edited and revised manuscript (LCH‐S, AC‐S, AH‐G, VM‐T, RC, DL and JAM), approved final version of manuscript (DL, AC‐S, JAM).

## FUNDING INFORMATION

This work was supported by personalized metacategorization of inflammatory processes associated with metabolic syndrome, autoimmune and viral diseases for precision medicine (METAINFLAMACIÓN, Ref: Y2020/BIO‐6600) funded by the Government of the Community of Madrid, Spain. The author AC‐S has received a research fellowship ‘Sara Borrell’ from the ‘Instituto de Salud Carlos III’ (CD22/00011).

## CONFLICT OF INTEREST STATEMENT

The authors declare that they have no conflicting interests.

## Supporting information


**FIGURE S1:** The horizontal axis is the fold difference of the difference species in the comparison group, while the vertical axis is the *p*‐value of the significant between‐group difference test for the difference species. Each point in the graph represents a differential bacterium, where up represents the higher abundance of that differential bacteria in the first comparison group than in the second comparison group, while down is the opposite. The position on the graph indicates how large and significant the expression difference is between the two compared diseases. Bacteria with significant and large changes appear in the upper regions of the plot (above a certain significance threshold) and are often labelled for quick identification.

## Data Availability

The data that support the findings of this research are totally available on request from the corresponding author (amanda.cuevas@alimentacion.imdea.org).
