# Supplementary material for: Comparative assessment of phenotypic markers in patients with chronic inflammation: Differences on Bifidobacterium concerning liver status
Source: Eur J Clin Invest. 2024 Oct 28;55(2):e14339. doi: 10.1111/eci.14339 (PMC11744921; doi:10.1111/eci.14339)

SUPPLEMENTAL MATERIAL

A volcano was performed to represent both the magnitude and statistical significance of differences between MI and SLE groups. The volcano plot shows the significantly up-regulated difference between bacteria and significantly down-regulated difference between groups of disease. This analysis showed that Bifidobacterium genus was upregulated in these participants.

Figure 1S. The horizontal axis is the fold difference of the difference species in the comparison group, while the vertical axis is the p-value of the significant between-group difference test for the difference species. Each point in the graph represents a differential bacterium, where up represents the higher abundance of that differential bacteria in the first comparison group than in the second comparison group, while down is the opposite. The position on the graph indicates how large and significant the expression difference is between the two compared diseases. Bacteria with significant and large changes appear in the upper regions of the plot (above a certain significance threshold) and are often labeled for quick identification.

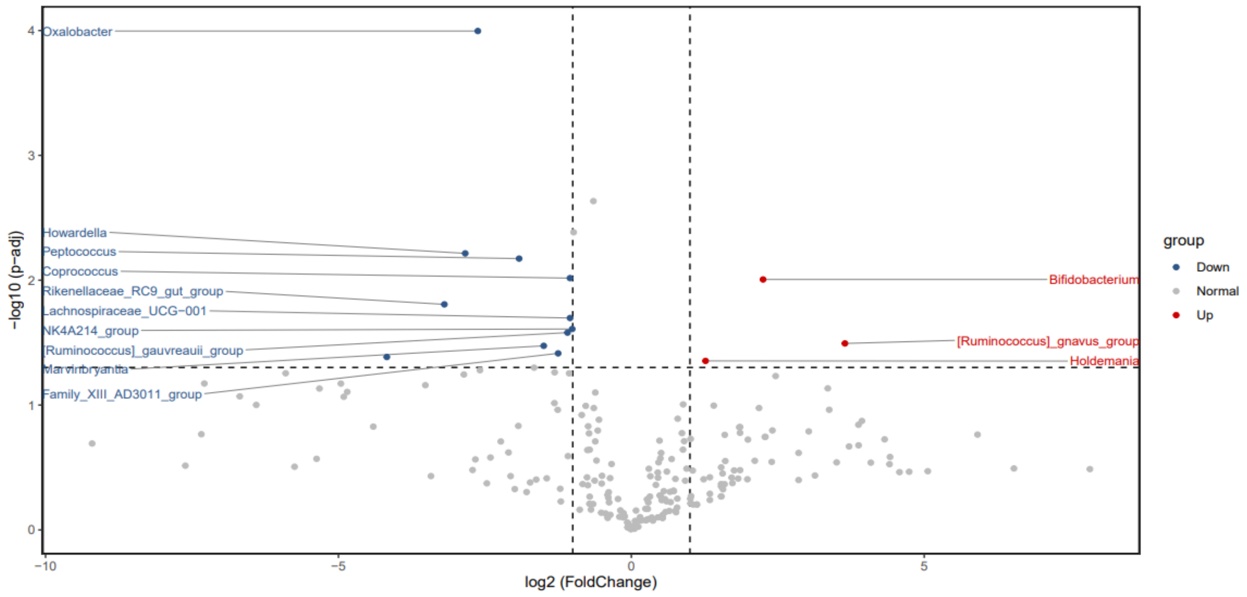

Supplement: Supplementary file 1 — FIGURE S1: The horizontal axis is the fold difference of the difference species in the comparison group, while the vertical axis is the p‐value of the significant between‐group difference test for the difference species. Each point in the graph represents a differential bacterium, where up represents the higher abundance of that differential bacteria in the first comparison group than in the second comparison group, while down is the opposite. The position on the graph indicates how large and significant the expression difference is between the two compared diseases. Bacteria with significant and large changes appear in the upper regions of the plot (above a certain significance threshold) and are often labelled for quick identification. [file ECI-55-e14339-s001.pdf]
